# *Lactobacillus plantarum* attenuates anxiety-related behavior and protects against stress-induced dysbiosis in adult zebrafish

**DOI:** 10.1038/srep33726

**Published:** 2016-09-19

**Authors:** Daniel J. Davis, Holly M. Doerr, Agata K. Grzelak, Susheel B. Busi, Eldin Jasarevic, Aaron C. Ericsson, Elizabeth C. Bryda

**Affiliations:** 1 Department of Veterinary Pathobiology, University of Missouri, Columbia, MO 65201, USA; 2 Center for Host-Microbial Interactions, Department of Biomedical Sciences, School of Veterinary Medicine, University of Pennsylvania, Philadelphia, PA 19104, USA; 3 University of Missouri Metagenomics Center (MUMC), University of Missouri, Columbia, MO 65201, USA

## Abstract

The consumption of probiotics has become increasingly popular as a means to try to improve health and well-being. Not only are probiotics considered beneficial to digestive health, but increasing evidence suggests direct and indirect interactions between gut microbiota (GM) and the central nervous system (CNS). Here, adult zebrafish were supplemented with *Lactobacillus plantarum* to determine the effects of probiotic treatment on structural and functional changes of the GM, as well as host neurological and behavioral changes. *L. plantarum* administration altered the β-diversity of the GM while leaving the major core architecture intact. These minor structural changes were accompanied by significant enrichment of several predicted metabolic pathways. In addition to GM modifications, *L. plantarum* treatment also significantly reduced anxiety-related behavior and altered GABAergic and serotonergic signaling in the brain. Lastly, *L. plantarum* supplementation provided protection against stress-induced dysbiosis of the GM. These results underscore the influence commensal microbes have on physiological function in the host, and demonstrate bidirectional communication between the GM and the host.

Stress and anxiety disorders are two of the most common psychiatric illnesses worldwide, affecting both children and adults. According to the American Psychiatric Association, people who experience stress and anxiety over long periods of time frequently experience deleterious health outcomes. Currently, the primary medical means to mitigate these conditions is pharmacological treatment that attempts to counteract the dysregulation of one or more major neurotransmitter systems in the brain associated with these disorders. These systems include the catecholaminergic (noradrenaline, dopamine), GABAergic (gamma-aminobutyric acid; GABA), glutamatergic (glutamate) and serotonergic (serotonin) systems^1^,^2^. However, these disorders not only manifest in specific behaviors and neurological symptoms, but are also often closely associated with various conditions affecting the digestive tract^3^. Despite sufficient evidence demonstrating the linkage between neurological and digestive disorders, current treatments focus on minimizing dysregulation at the neurological level. Moreover, the efficacy of anxiolytic compounds used to treat anxiety disorders varies between individuals^4^, and new approaches to treat these conditions are needed.

Notably, manipulation and regulation of an organism’s gut microbiota (GM) may provide an alternative or adjunct to current approaches. Probiotics have become increasingly popular as a means to improve health and well-being. Not only are probiotics considered beneficial to digestive health, but increasing evidence of direct and indirect interactions between the GM and the central nervous system (CNS) suggests beneficial effects to neurological health as well. Specifically, in addition to direct effects on the innate immune system and composition of the resident microbiota, certain strains of *Lactobacillus* exert a positive effect on anxiety-related behavior and responses to stress^5^,^6^.

Zebrafish (*Danio rerio*) are an emerging model species for neurobehavioral studies and their use is well-established in the drug screening sector. Zebrafish exhibit numerous behaviors that have been correlated with those seen in human neurological processes and disorders, such as anxiety^7^, learning^8^, fear^9^, sociability^10^, and psychosis^11^, and zebrafish neurotransmitter systems demonstrate clear translatability to other organisms including rodents and humans^12^. Moreover, studies utilizing anxiolytic and anxiogenic treatments have validated many anxiety-related behavioral tests in zebrafish models both qualitatively and quantitatively^13^. While probiotics have been shown to modulate innate immunity and expression of stress-related genes in zebrafish^14^, it is currently unknown whether there is a corresponding reduction in stress- and anxiety-related behavior in the fish. Additionally, the effect of probiotics on the structure and function of the GM is not well understood in zebrafish.

Here, 16S rRNA amplicon sequencing was performed on DNA extracted from the intestinal content of zebrafish with and without *L. plantarum* supplementation. Furthermore, *L. plantarum* treatment was also correlated with anxiety-related behavioral testing in a novel tank diving test. In conjunction with behavioral changes, gene regulation involved in the GABAergic and serotonergic pathways were analyzed in the brains of *L. plantarum*-treated and non-treated zebrafish. Lastly, the influence of *L. plantarum* on stress-induced changes in the composition of the GM were evaluated. These data characterize the effect *L. plantarum* has on GM structure and predicted function in adult zebrafish. Moreover, these data illustrate the ability of *L. plantarum* to attenuate anxiety-related behavior and stress-induced dysbiosis of the GM.

## Results

### Zebrafish GM structure and function is altered by *L. plantarum* treatment

**Figure 1:**
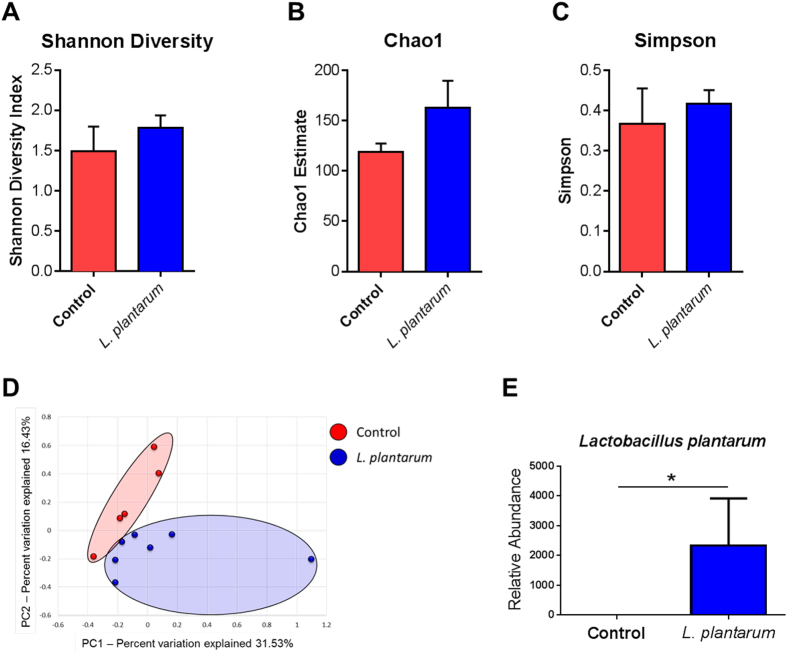
*L. plantarum* supplementation alters the structure of the GM in adult zebrafish. (**A–C**) *L. plantarum* treatment did not alter alpha diversity of the GM as determined by (**A**) Shannon Diversity Index, (**B**) Chao1 Index, and (**C**) Simpson Index. (**D**) Principal component analysis demonstrates distinct structural differences in the GM of zebrafish treated with *L. plantarum* compared to controls. (**E**) qPCR confirms a significant elevation of *L. plantarum* species in treated zebrafish. Data shown are represented by mean ± SEM (*n* = 5–7 fish/group for 16S rRNA sequencing data and *n* = 5–7 fish/group analyzed in duplicate for qPCR data). Asterisks (*) denote *p* values ≤ 0.05 (student’s *t*-test).

**Figure 2:**
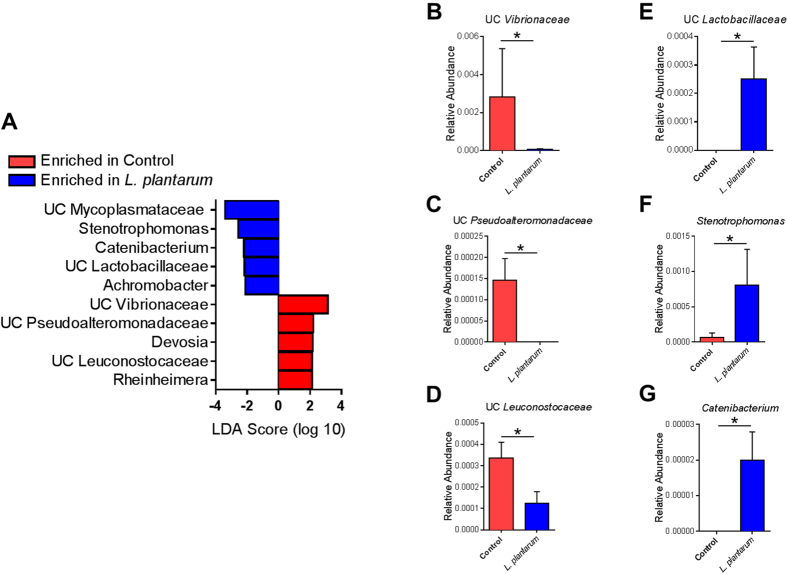
LEfSe analysis reveals minor core changes in the GM of *L. plantarum* treated zebrafish. (**A**) Linear discriminant analysis (LDA) effect size (LEfSe) revealed specific taxa enriched in either the control (red) or *L. plantarum*-treated (blue) groups. (**B–G**) Relative abundance of taxa found to be significantly altered by *L. plantarum* treatment. Data shown are represented by mean ± SEM (*n* = 5–7 fish/group). Asterisks (*) denote *p* values ≤ 0.05 (student’s *t*-test).

Comparison of the detected microbial profiles at the taxonomic level of phylum did not reveal any major core shifts of the GM (Supp. Table 1). Similar to previous reports^15^, zebrafish GM was dominated by the phyla *Fusobacteria* (mean ± SEM relative abundance of 76.9 ± 8.3%) and *Proteobacteria* (11.9 ± 5.2%). *L. plantarum* treatment did not significantly alter the α-diversity, as determined via the Shannon diversity index (Fig. 1A), Chao1 index (Fig. 1B), or the Simpson diversity index (Fig. 1C). Moreover, principal component analysis (PCA) revealed that while there was subtle separation of groups PERMANOVA failed to detect a significant difference in β-diversity (Fig. 1D). A *L. plantarum* species-specific qPCR was performed to confirm successful colonization of *L. plantarum* in the zebrafish gut (Fig. 1E). Next, linear discriminant analysis (LDA) effect size (LEfSe) was used to determine microbial alterations that were significantly associated with *L. plantarum* treatment^16^. It was found that unclassified (UC) *Vibrionaceae*, UC *Pseudoalteromonadaceae*, *Devosia*, UC *Leuconostocaceae*, and *Rheinheimera* were significantly enriched in the control group, whereas UC *Mycoplasmataceae*, *Stenotrophomonas*, *Catenibacterium*, UC *Lactobacillaceae*, and *Achromobacter* were enriched in the *L. plantarum*-treated group (Fig. 2A–G).

**Figure 3:**
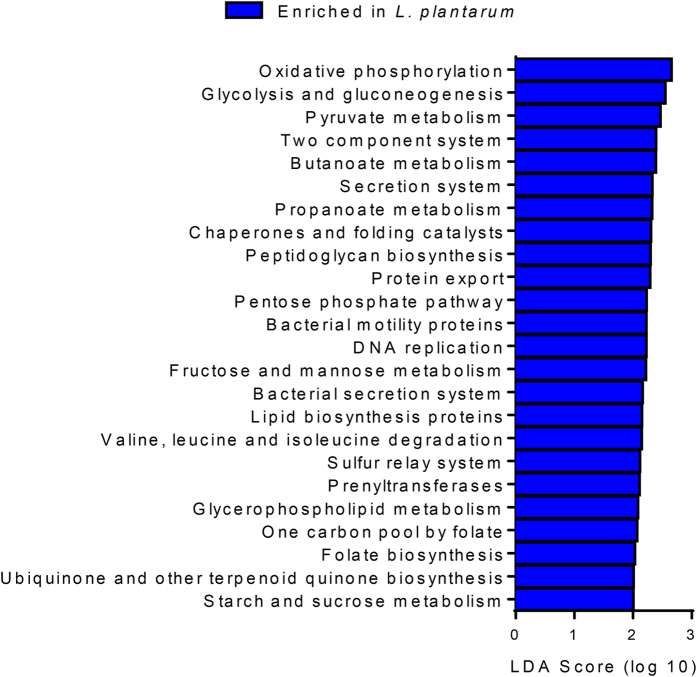
PICRUSt analysis reveals functional changes in the GM of *L. plantarum* treated zebrafish. Phylogenetic Investigation of Communities by Reconstruction of Unobserved States (PICRUSt) predicted functional profile alteration of microbial communities enriched in *L. plantarum* treated zebrafish. (*n* = 5–7 fish/group). (Linear discriminant analysis; LDA).

To determine the metabolic alterations associated with the GM differences, the Phylogenetic Investigation of Communities by Reconstruction of Unobserved States (PICRUSt) software package was applied to the current 16S rRNA amplicon dataset^17^. A total of 24 different predicted gene functions, grouped according to KEGG (Kyoto Encyclopedia of Genes and Genomes) category^18^, were predicted to be significantly enriched in the *L. plantarum* treated zebrafish (Fig. 3). Most of these pathways are broadly involved in energy metabolism and vitamin biosynthesis.

### *L. plantarum* alters anxiety-related behavior and the serotonergic pathway in adult zebrafish

**Figure 4:**
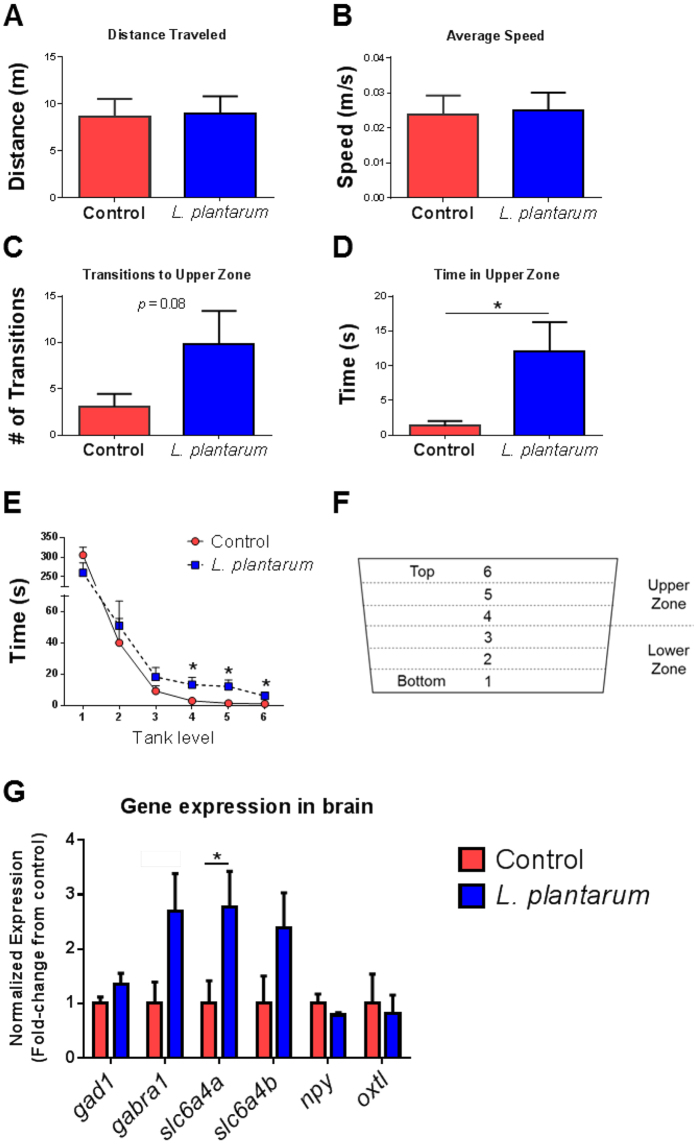
*L. plantarum* modulates anxiety-related behavior and the GABAergic pathway in adult zebrafish. (**A**,**B**) No differences were detected in locomotor activity between controls and *L. plantarum* treated zebrafish. (**C**‒**F**) Zebrafish supplemented with *L. plantarum* exhibit significantly less anxiety-related behavior than controls as indicated by number of transitions into the upper zone (**C**) and time spent in the upper portions of the tank (**D**‒**F**). (**G**) Quantitative real-time PCR of candidate genes thought to be associated with anxiety-related behavior. Genes encoding the GABA-A alpha 1 receptor (*gabra1*) and serotonin transporter A (*slc6a4a*) are up-regulated in *L. plantarum* treated zebrafish. No differences were detected in neuropeptide expression levels (*npy* and *oxtl*) in *L. plantarum* treated fish compared to controls. Data shown are represented by mean ± SEM (*n* = 14–16 fish/group for behavior data and *n* = 6 fish/group performed in duplicate for qPCR data). Asterisks (*) denote *p* values ≤ 0.05 (student’s *t*-test).

Novel tank diving is a validated behavior test for assessing anxiety-related behavior in adult zebrafish, wherein the time spent in top portions of the tank correlates with less anxiety. To determine if *L. plantarum* influences anxiety-related behavior in adult zebrafish, a novel tank diving test was performed on fish supplemented with and without *L. plantarum*. Zebrafish from both groups displayed similar locomotor activity (*p* > 0.05) as measured by the total distance traveled and average swimming speed (Fig. 4A,B). However, zebrafish supplemented with *L. plantarum* exhibited a strong trend of more transitions to the upper zone (*p* = 0.08) and spent significantly more time in the upper portion of the tank (*p* = 0.01) (Fig. 4C–F). These results suggest that *L. plantarum* is sufficient in reducing anxiety-related behavior in adult zebrafish.

To assess the expression levels of genes associated with anxiety-related behavior, quantitative real-time PCR was performed on samples extracted from the brains of control and *L. plantarum* treated zebrafish. The gene encoding for the GABA-A receptor alpha 1, *gabra1*, had a trend of up-regulation in *L. plantarum*-treated fish (*p* = 0.06). Glutamic acid decarboxylase (*gad1*) also had a trend in *L. plantarum* treated zebrafish (*p* = 0.13) (Fig. 4G). Similarly, genes encoding for serotonin transporters (*slc6a4a* and *slc6a4b*) were also up-regulated in *L. plantarum*-treated zebrafish, although *slc6a4b* was not statistically significant (*p* = 0.04 and 0.12, respectively) (Fig. 4G). Neuropeptides also have modulatory roles in a variety of behaviors including stress and anxiety. However, *L. plantarum* treatment had no effect on neuropeptide Y (*npy*) and isotocin (*oxtl*) expression levels (*p* > 0.05) (Fig. 4G). Collectively, these results suggest that the anxiolytic effect of *L. plantarum* could be due to modulation of GABAergic and/or serotonergic pathways.

### Elevation of serum cortisol and leukocyte patterning in response to a chronic unpredictable stress (CUS) test is unaffected by *L. plantarum* treatment

**Figure 5:**
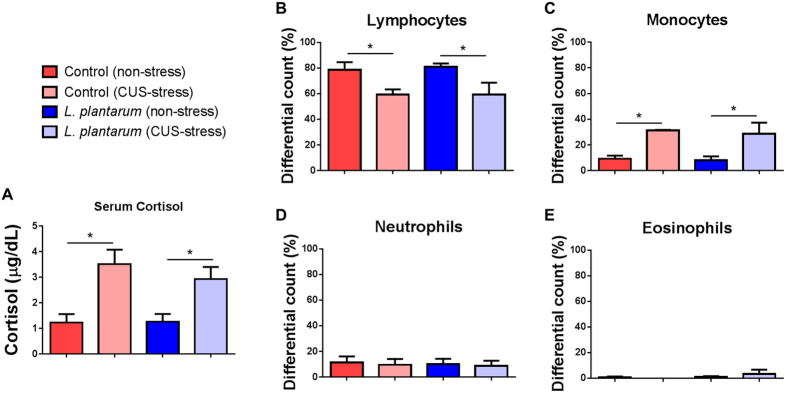
Physiological effects of CUS are unaffected by *L. plantarum* treatment. (**A**) Serum cortisol was significantly elevated in zebrafish subjected to CUS. (**B**‒**E**) Leukocyte differentials reveal that CUS zebrafish have a significant reduction of circulating lymphocytes (**B**), a significant increase in circulating monocytes (**C**), and no alterations in neutrophils or eosinophils (**D**,**E**, respectively) compared to non-stressed controls. Data shown are represented by mean ± SEM (*n* = 12 fish/group for ELISA data and *n* = 3‒4 fish/group for leukocyte differentials). Asterisks (*) denote *p* values ≤ 0.05 (two-way ANOVA).

Downstream events from activation of the hypothalamic pituitary interrenal (HPI) axis were examined in order to determine if *L. plantarum* modified physiological effects of CUS. Serum cortisol levels were significantly elevated (*p* < 0.05) as a result of CUS in both the control and *L. plantarum*-supplemented groups (Fig. 5A). Cortisol levels from CUS fish treated with *L. plantarum* were not found to be significantly lower than those of the CUS control group (Fig. 5A).

Increased steroid hormones in the plasma often lead to characteristic stress leukogram patterns. Leukocyte differentials were performed to determine whether *L. plantarum* treatment led to altered leukogram patterns. CUS was found to significantly decrease the amount of circulating lymphocytes (*p* < 0.01) in both control and *L. plantarum*-supplemented fish (Fig. 5B). A monocytosis was also revealed in zebrafish subjected to CUS independent of *L. plantarum* treatment (*p* < 0.01) (Fig. 5C). No effect was observed on neutrophil and eosinophil counts (Fig. 5D,E). Taken together, these results suggest that *L. plantarum* supplementation is insufficient in attenuating effects of HPI axis activation due to CUS in adult zebrafish.

### Stress-induced dysbiosis of the GM is protected by *L. plantarum* in adult zebrafish

**Figure 6:**
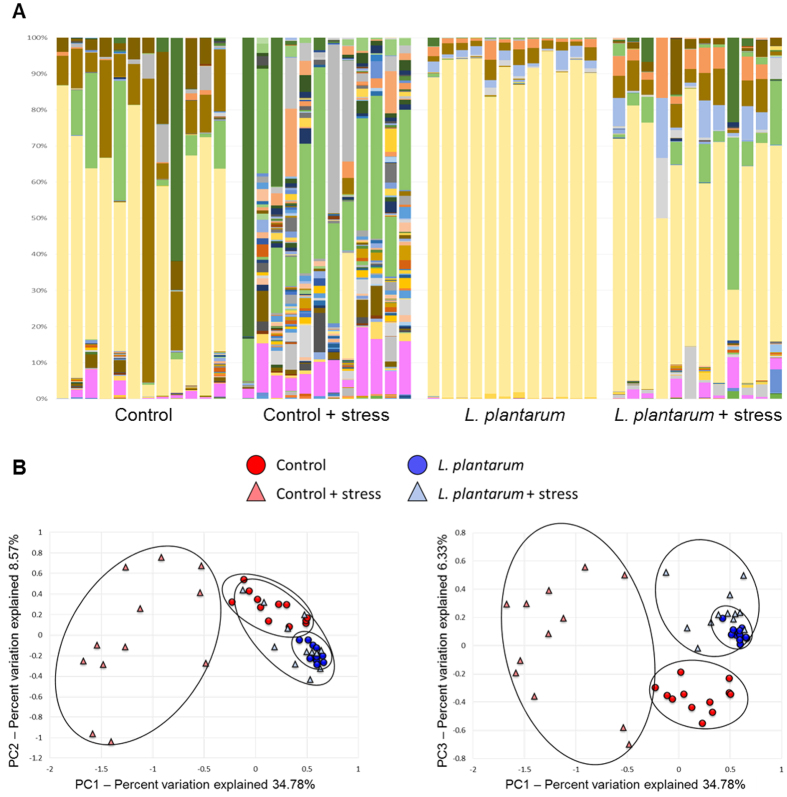
Stress-induced shifts in the GM are attenuated by *L. plantarum*. (**A**‒**C**) Chronic unpredictable stress (CUS) induced dramatic alterations of the GM in control zebrafish, however *L. plantarum* treatment was shown to be protective against this stress-induced dysbiosis. (**A**) The relative abundance of the core phylum, *Fusobacteria* (indicated by the yellow bar), was greatly diminished in the chronically stressed control group, whereas the major core GM remained intact in the chronically stressed *L. plantarum* treated fish. (**B**,**C**) Principal component analysis revealed significant shifts in response to CUS in the control group, while chronically stressed *L. plantarum* treated zebrafish cluster in conjunction with non-stressed *L. plantarum* treated fish. (*n* = 12 fish/group).

To assess the influence of CUS on the architecture of the GM, 16S rRNA amplicon sequencing was performed on stressed and non-stressed zebrafish. This was done on both control fish and fish supplemented with *L. plantarum* to determine if probiotic treatment had any interactions with stress-induced GM changes. Zebrafish not supplemented with *L. plantarum* exhibited dramatic shifts in the GM after CUS. This stress-induced GM shift resulted in diminishment of the relative abundance of the core phylum, *Fusobacteria* (*p* < 0.001). However, the major core GM remained intact in zebrafish treated with *L. plantarum* after a chronic stress treatment (Fig. 6A). A complete list of OTUs altered by CUS exposure can be found in Supplementary Table 2. Principal component analysis further demonstrated a significant shift in the GM structure in chronically stressed zebrafish not supplemented with *L. plantarum* (Fig. 6B,C). Analysis via MANOVA detected significant main effects of both CUS and treatment with *L. plantarum* (*p* = 0.018 for both), as well as a significant interaction between those variables (*p* = 0.017). Overlaid clustering between the stressed and non-stressed groups indicated that no major alterations occurred in the GM structure of chronically stressed zebrafish supplemented with *L. plantarum* (Fig. 6B,C). Additionally, *L. plantarum* protected from particular stress-induced KEGG orthologue pathway up- and down- regulation. Riboflavin and other vitamin biosynthesis and metabolism was significantly downregulated in stressed zebrafish not supplemented with *L. plantarum* (Supp. Fig. 1). Enrichment of these specific pathways remained unaltered in *L. plantarum*-treated fish subjected to CUS (Supp. Figs 2 and 3). These results suggest that stress-induced dysbiosis (both structurally and functionally) of the GM can be mitigated by *L. plantarum* treatment in zebrafish.

## Discussion

The current data provide evidence that probiotic treatment alters the structure and function of the GM and mitigates behavioral responses in adult zebrafish. Specifically, this study shows that *L. plantarum* administration causes subtle alterations of the GM and enriches a number of KEGG pathway orthologues involved in energy production. Furthermore, *L. plantarum* treatment was shown to significantly reduce anxiety-related behavior and modulate serotonergic signaling in the brain. GABAergic signaling was also thought to be altered in the *L. plantarum* treated fish, however, no statistically significant results were found. The lack of significant findings is likely due to the large variance of gene expression found in individual zebrafish and the modest sample size analyzed. Effects of HPI axis activation in response to chronic stress was not mitigated by *L. plantarum*. However, the GM structure displayed a significant shift in response to a chronic stressor in control fish and *L. plantarum* treatment was protective against this stress-induced dysbiosis of the GM. These data are a further indication of the bidirectional communication between the GM and the host, as well as additional validation for the use of zebrafish as a model for gut-brain interactions.

Previous studies have shown the microbiota of most mammals is dominated by the phyla *Firmicutes* and *Bacteroidetes*, whereas the core microbiota of zebrafish predominantly consists of bacteria from the *Proteobacteria* and *Fusobacteria* phyla^15^,^19^,^20^. However, studies have also shown similarities between the zebrafish microbiota and that of rodents and humans, such as colonization by species from the genus *Lactobacillus*^14^,^21^,^22^. The data shown here are congruent with these other findings, since we found that the GM of healthy adult zebrafish primarily consists of *Fusobacteria* and administration of *L. plantarum* does indeed result in colonization in the gut. To date, there are a limited number of studies demonstrating the associations of altered GM following treatment with probiotics. The data reported here show that administration of *L. plantarum* did not alter α-diversity of the GM and the GM remains dominated by the phyla *Proteobacteria* and *Fusobacteria*. However, principal component analysis (PCA) illustrating β-diversity revealed distinct clustering of control fish and fish treated with *L. plantarum*. These minor alterations are not completely surprising given that other studies have shown that administration of multiple probiotics can significantly alter the GM^23^,^24^. Specifically, *Lactobacillus rhamnosus* modulates the GM associated with a reduction of serum lipids in the hyperlipidemic rat^23^. Moreover, it has been reported that *Lactobacillus reuteri* supplementation alters the oral microbiota within a 12 week period^24^.

To determine if functional metabolic changes accompanied *L. plantarum*-induced GM shifts, the 16S rRNA amplicon dataset was subjected to Phylogenetic Investigation of Communities by Reconstruction of Unobserved States (PICRUSt) analysis. Recent human microbiome studies have shown that alterations in the GM due to *Clostridium difficile* colonization significantly alter metabolic pathways involved in amino acid biosynthesis and transport and binding carbohydrates^25^. The results of the current study further illustrate how GM modifications can influence metabolomics. *L. plantarum* administration significantly enriched many metabolic pathways involved in energy metabolism and vitamin biosynthesis. Notably, folate (folic acid) biosynthesis was predicted to be enriched with *L. plantarum* supplementation. This is congruent with previous studies showing the ability of probiotics to synthesize vitamins such as folic acid, riboflavin, and vitamin B_12_^26^,^27^. Interestingly, both riboflavin and folic acid have been correlated with protection against behavioral impairments and stress^28^,^29^.

Many nutrients such as vitamins, amino acids, or dietary fibers that are consumed by the host are assimilated and converted into other metabolites by microbes within the gastrointestinal tract. Products of these biochemical conversions, such as short-chain fatty acids (SCFAs), biogenic amines, or other amino acid-derived compounds such as serotonin or GABA, may be biologically active within the host^30^. Previous studies have shown that many of these neuroactive compounds that can be produced by the GM have the ability to alter neurological function and behavior^31^. One particular pathway of communication between the GM and the CNS is mediated via GABA transmission and is dependent upon the vagus nerve^6^. Our data provide further evidence of GM signaling through the GABAergic pathway to alter behavior. Specifically, zebrafish treated with *L. plantarum* exhibited significantly less anxiety-related behavior compared to untreated controls. Along with behavioral modulation, *L. plantarum* administration also correlated with a trending increase of GABA-A receptor and significant increase in serotonin transporter expression in the brain. These findings are consistent with a recent study that shows up-regulation of genes involved in serotonergic signaling in the brain of *L. rhamnosus-*treated zebrafish^32^. Although no direct causative relationship was investigated in this study, it is known that regulation of GABAergic and serotonergic pathways are associated with anxiety-related behavior in many species^33^,^34^,^35^,^36^,^37^. Many of the current therapeutics available for treating anxiety-related symptoms are serotonin reuptake inhibitors (SSRIs) and benzodiazepines. The primary mechanism of action of these drugs is to increase serotonin and GABA transmission in the brain, respectively. Moreover, it has been shown that some strains of *Lactobacillus* catalyze the decarboxylation of glutamate, resulting in the production of GABA and CO_2_^38^.

Anxiety and anxiety-related behavior is frequently accompanied with activation of the HPA axis and stress. Furthermore, it has been shown that GM modulation of anxiety-related behavior often correlates with attenuation of stress responses in rodents^6^,^39^. To test the ability of *L. plantarum* to attenuate responses to stress, zebrafish with and without *L. plantarum* supplementation were subjected to a chronic unpredictable stress (CUS) paradigm. Responses to stress rely heavily on the hypothalamic-pituitary-adrenocortical (HPA) axis and its synthesis of glucocorticoids. Previous reports have demonstrated functional and anatomical parallels between the zebrafish hypothalamic-pituitary-interrenal (HPI) axis and the mammalian HPA axis, wherein cortisol is the primary corticosteroid produced in both zebrafish and humans^40^,^41^,^42^. During a characteristic stress response, corticosteroids often modulate leukocyte trafficking in many species^43^,^44^,^45^. Typically, a stress leukogram consists of a marginalization of lymphocytes, complemented by an increase in serum monocytes and neutrophils. Results shown here do not recapitulate these findings in adult zebrafish. Activation of the HPI axis was shown in both the control group and fish supplemented with *L. plantarum* in response to a chronic stress paradigm. Serum cortisol was significantly elevated in all fish subjected to CUS independent of *L. plantarum* supplementation. The elevation of serum cortisol was also accompanied by a significant lymphopenia and monocytosis in all stress groups. The discrepancies between probiotics modulating stress responses in rodents and seemingly not affecting HPI axis activation in our study are possibly due to the nature of chronic unpredictable stressor administered.

Probiotics are often used to correct dysbiotic states of the GM. The results of the current study demonstrate that probiotic supplementation is protective against stress-induced dysbiosis of the GM in adult zebrafish. Specifically, the major core phylum (*Fusobacteria*) was greatly diminished in chronically stressed zebrafish not treated with *L. plantarum*. However, only minor stress-induced changes occurred in zebrafish with probiotic supplementation. Microbiota structural alterations were accompanied by predicted functional metabolic changes. In particular it was shown that stress-induced downregulation of riboflavin biosynthesis occurred in stressed fish not supplemented with *L. plantarum*. Pathways involved in synthesis of riboflavin and other vitamin were of particular interest due to their protective roles against oxidative stress^29^. In conclusion, this study demonstrates that probiotics modify the GM as well as additional evidence of key metabolic pathways that could lead to physiological changes in the host. These findings underscore the influence of commensal microbes on neurological function and behavioral responses, while demonstrating clear bidirectional communication between the GM and the host. Moreover, the results provide further support for the use of zebrafish for microbiota-related neuroimmune research.

## Methods

### Animals

Adult wild-type zebrafish were purchased from Aquatica BioTech (Sun City Center, FL) and acclimated to the facility for 1 week prior to any treatment. Zebrafish were maintained in recirculating 3 L tanks at 28 ± 2 °C on a 14:10 h light:dark cycle and fed commercial fish diet.

*Lactobacillus* treatment groups were supplemented with 1 × 10^6^ CFU/mL of *Lactobacillus plantarum* (USDA-ARS, Washington DC) twice a day for one month prior to behavior testing or microbiota analysis. *L. plantarum* was administered according to adaptations of previous studies via supplementing the tank water during feeding times^46^,^47^,^48^. A chronic unpredictable stress (CUS) paradigm was modified from a previous study^49^. Specific conditions for the multiple stressors can be found in Supplementary Table 3. All experimental procedures were approved by the University of Missouri’s Institutional Animal Care and Use Committee and were performed according to the guidelines set forth in the Guide for the Use and Care of Laboratory Animals.

### Microbial DNA extraction and quantification

Microbial DNA was extracted according to a previously published protocol optimized for microbiota analysis in adult zebrafish^15^. Immediately following euthanasia, zebrafish GI tracts were aseptically collected into 800 μL of lysis buffer (500 mM NaCl, 50 mM tris-HCl, 50 mM EDTA, and 4% SDS), homogenized for 3 minutes in a Qiagen Tissuelyser II, and incubated at 70 °C for 20 minutes. Following centrifugation at 5000 × g for 5 minutes at room temperature, the supernatant was mixed with 200 μL of 10 mM ammonium acetate, incubated on ice for 5 minutes, and then centrifuged at 16,000 × g for 10 minutes at room temperature. 750 μL of supernatant was then mixed with an equal volume of chilled isopropanol, and incubated for 30 minutes on ice. The contents of the tube were then centrifuged at 4 °C for 15 minutes to pellet DNA. The pellet was rinsed twice with 70% EtOH and re-suspended in 150 μL of tris-EDTA. Fifteen μL of proteinase-K and 200 μL of buffer AL (DNeasy kit, Qiagen) were added and tubes were incubated at 70 °C for 10 minutes. 200 μL of 100% EtOH was then added and the entire contents of the tube were transferred to a Qiagen spin column before continuing with the manufacturer’s instructions for DNA purification (DNeasy Kit, Qiagen). DNA was eluted in 200 μL of EB buffer (Qiagen). Yield of double-stranded DNA was determined via fluorometry (Qubit 2.0, Life Technologies, Carlsbad, CA) using Qubit^®^ dsDNA BR assay kits (Life Technologies).

### Metagenomic library preparation and sequencing

Sequencing of the V4 region of the 16S rRNA gene was performed on the Illumina MiSeq platform. Bacterial 16S ribosomal DNA amplicons were constructed by amplification of the V4 hypervariable region of the 16S rRNA gene with primers flanked by Illumina standard adapter sequences. Universal primers (U515F/806R) previously developed against the V4 region were used for generating amplicons. Oligonucleotide sequences were obtained at proBase. A single forward primer and reverse primers with unique 12-base indices were used in all reactions. Extracted DNA was quantitated by Qubit flourometer using the quant-iT HS dsDNA reagent kit (Invitrogen). PCR reactions (50 uL) contained 100 ng of genomic DNA, forward and reverse primers (0.2 uM each), dNTPs (200 uM each), and Phusion High-Fidelity DNA Polymerase (1 U). PCR amplification was performed as follows: amplification at 98 °C for 3 minutes, and 25 cycles at 98 °C for denaturation for 15 seconds, annealing at 50 °C for 30 seconds, and extension at 72 °C for 30 seconds, then a final extension at 72 °C for 7 minutes. Amplified product (5 ul) from each reaction was combined and thoroughly mixed; pooled amplicons were purified by addition of Axygen AxyPrep MagPCR Clean-up beads (50 uL) to an equal volume of 50 uL of amplicons and incubated at room temperature for 15 minutes. Products were washed multiple times with 80% ethanol and the dried pellet resuspended in Qiagen EB Buffer (32.5 uL), incubated at room temperature for 2 minutes, and then placed on a magnetic stand for 5 minutes. Supernatant (30 uL) was transferred to a low-binding microcentrifuge tube for storage. The final amplicon pool was evaluated using the Advanced Analytical Fragment Analyzer automated electrophoresis system, quantified with the Qubit flourometer using the quant-iT HS dsDNA reagent kit, and diluted according to the manufacturer’s protocol for sequencing on the MiSeq.

### Bioinformatic analysis

Assembly, binning, and annotation of DNA sequences were performed at the MU Informatics Research Core Facility (IRCF). Briefly, contiguous sequences of DNA were assembled using FLASH software^50^ and contigs were culled if found to be short after trimming for a base quality less than 31. Qiime v1.7^51^ software was used to perform de novo and reference-based chimera detection and removal, and remaining contigs were assigned to operational taxonomic units (OTUs) using a criterion of 97% nucleotide identity. Taxonomy was assigned to selected OTUs using BLAST^52^ against the Greengenes database^53^ of 16S rRNA sequences and taxonomy.

### Quantitative RT-PCR

To measure the mRNA abundance of *gad1*, *gabra1*, *slc6a4a*, *slc6a4b*, neuropeptide Y (*npy*), and isotocin (*oxtl*), zebrafish brains were removed immediately following euthanasia. RNA was extracted from individual brains via an RNeasy kit (Qiagen) and cDNA synthesized using an EasyScript Plus™ cDNA Synthesis kit (Lambda Biotech, Ballwin, MO). Samples were then analyzed in duplicate and target mRNA expression was normalized to *ef1a* (housekeeping gene) expression. A complete list of primer sequences and references can be found in Supplementary Table 4. Every 10 μL reaction contained 1× SsoAdvanced universal SYBR^®^ Green supermix (BioRad, Hercules, CA), 0.3 μM forward and reverse primers, and 100 ng cDNA template. PCR parameters were: amplification at 95 °C for 3 minutes, and 50 cycles of denaturation at 95 °C for 15 seconds, annealing at 60 °C for 20 seconds, and extension at 72 °C for 20 seconds, with a plate read after each cycle (CFX384™ real-time system, BioRad).

### Novel tank diving

Anxiety-related behavior was assessed by subjecting zebrafish to a novel tank diving test as previously described^54^. Novel tank diving is a validated assessment of anxiety in zebrafish that has been extensively studied using anxiolytic and anxiogenic drugs that significantly attenuate and enhance this behavior, respectively^54^. Briefly, fish were individually placed into a novel tank and allowed to freely explore the tank for 6 minutes. ANY-maze video tracking software (Stoelting, Wood Dale, IL) was used to measure distance traveled, speed, transitions to various levels of the tank and time spent within those levels. The water was changed and tanks cleaned in between each trial to eliminate any alarm cues from previously tested fish.

### Serum collection and cortisol ELISA

Euthanasia and serum collection was performed using previously described techniques^55^. Each fish was placed into an individual 50-mL conical tube containing 20 mL of sterile water with 0.1% (v/v) of clove oil. Blood was collected by cutting off the tail three millimeters cranial to the caudal fin. Fish were then placed in a fenestrated microtube (0.6 mL) nested within a 1.5 mL microfuge tube for centrifugation at 400 × g for 5 min at room temperature. The 1.5 mL tubes containing the blood samples were centrifuged at 4 °C, 13,800 × g for 15 min. Supernatants were recovered and the serum was stored at −80 °C until analysis. Serum cortisol concentrations were determined using a cortisol ELISA kit (Salimetrics, Carlsbad, CA) according to the manufacturer’s instructions. The sensitivity of the assay is less than 0.007 μg/dL, and cortisol concentrations were read on a plate reader (SpectraMax M3, Molecular Devices, Sunnyvale, CA).

### Leukocyte differentials

Following euthanasia, blood was collected into a heparinized capillary tube via cardiac puncture. Blood smears were immediately prepared from whole blood and stained with Wright-Giemsa using a Hematek Slide Stainer (Siemens Health Care Diagnostics Inc., Tarrytown, NY). The slides were examined by light microscopy and leukocyte differentials were performed under oil immersion with a 100× objective.

### Statistics

Data were analyzed using Prism (GraphPad Software) and SigmaPlot (Systat Software, San Jose, CA). Statistical significance was set at a *p* value of less than 0.05. Principal component analysis was performed using a non-linear iterative partial least squares algorithm implemented in an Excel macro kindly provided by Hiroshi Tsugawa of the Riken Institute (Wako, Japan) to evaluate β-diversity and its association with zebrafish treatment. MANOVA was performed using SPSS Amos 23 (IBM, Armonk, NY). PERMANOVA was performed using the Past 3.x software package, freely available at folk.uio.no/ohammer/past/. A student’s *t*-test was used for α-diversity indexes, relative microbial abundance data, behavior analysis, and for gene expression. Leukocyte counts and cortisol data were analyzed using a two-way ANOVA, with probiotic treatment as one factor and stress as the second factor. A student-newman-keuls post-hoc test was performed on ANOVA analyses.

## Additional Information

**How to cite this article**: Davis, D. J. *et al*. *Lactobacillus plantarum* attenuates anxiety-related behavior and protects against stress-induced dysbiosis in adult zebrafish. *Sci. Rep.* **6**, 33726; doi: 10.1038/srep33726 (2016).

## Supplementary Material

Supplementary Information

Supplementary Dataset 1

Supplementary Dataset 2
