# Supplementary material for: Lactobacillus plantarum attenuates anxiety-related behavior and protects against stress-induced dysbiosis in adult zebrafish
Source: Sci Rep. 2016 Sep 19;6:33726. doi: 10.1038/srep33726 (PMC5027381; doi:10.1038/srep33726)
Supplement: Supplementary Information [file srep33726-s1.pdf]

***Lactobacillus plantarum* attenuates anxiety-related behavior and protects against stress-induced dysbiosis in adult zebrafish**

Daniel J. Davis[1#], Holly M. Doerr[1#], Agata K. Grzelak[1], Susheel B. Busi[1], Eldin Jasarevic[2], Aaron C. Ericsson[1,3], and Elizabeth C. Bryda[1,]*

[1] Department of Veterinary Pathobiology, University of Missouri, Columbia, MO 65201, USA
[2] Center for Host-Microbial Interactions, University of Pennsylvania, Philadelphia, PA 19104, USA
[3] University of Missouri Metagenomics Center (MUMC), University of Missouri, Columbia, MO 65201, USA

*Correspondence: brydae@missouri.edu

# Co-first authors

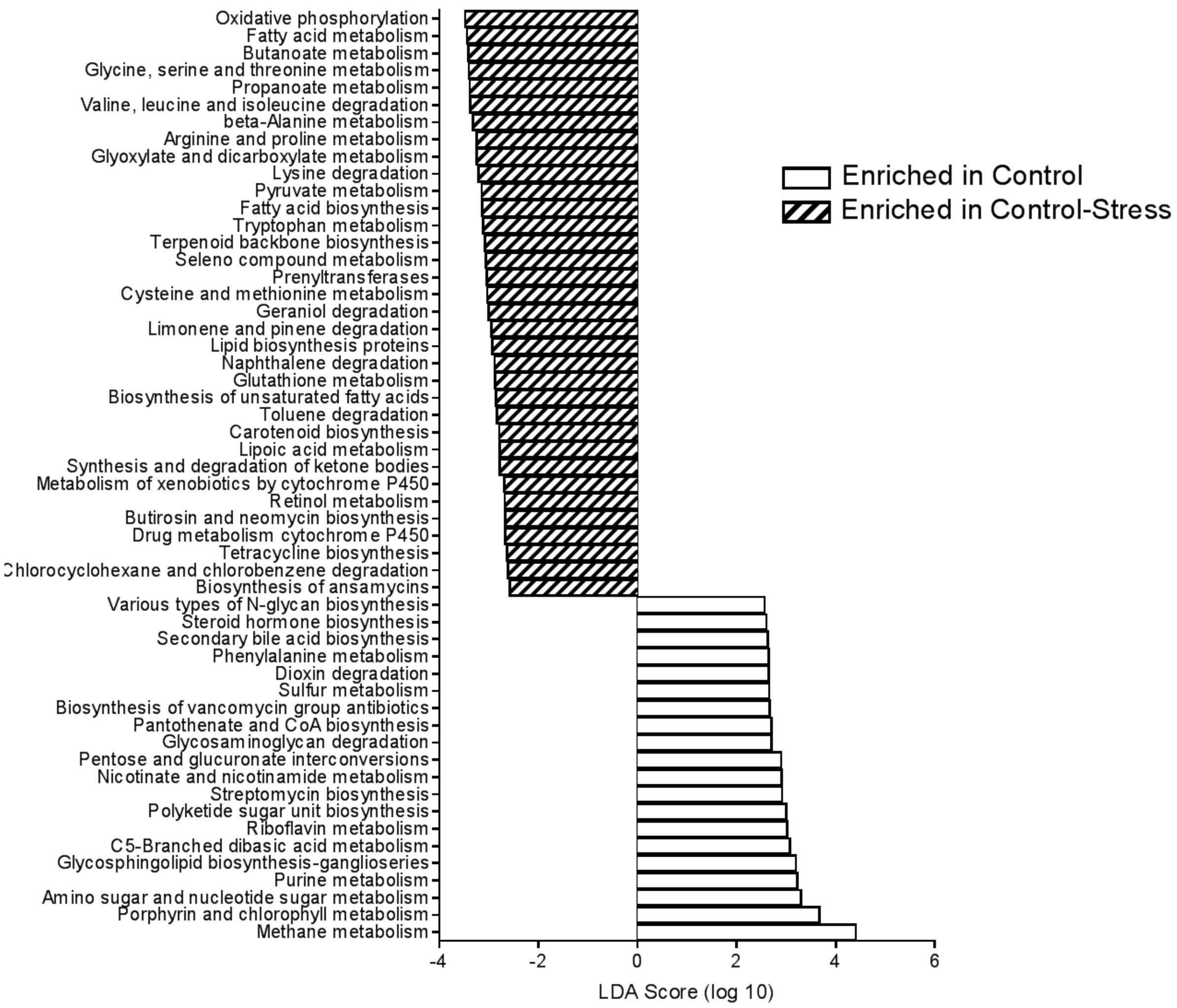

Supplementary Figure 1 **PICRUSt analysis reveals stress-induced functional alterations of the GM.** Phylogenetic Investigation of Communities by Reconstruction of Unobserved States (PICRUSt) predicted functional profile alteration of microbial communities in control zebrafish subjected to chronic unpredictable stress (CUS). (*n* = 12 fish/group). (Linear discriminant analysis; LDA).

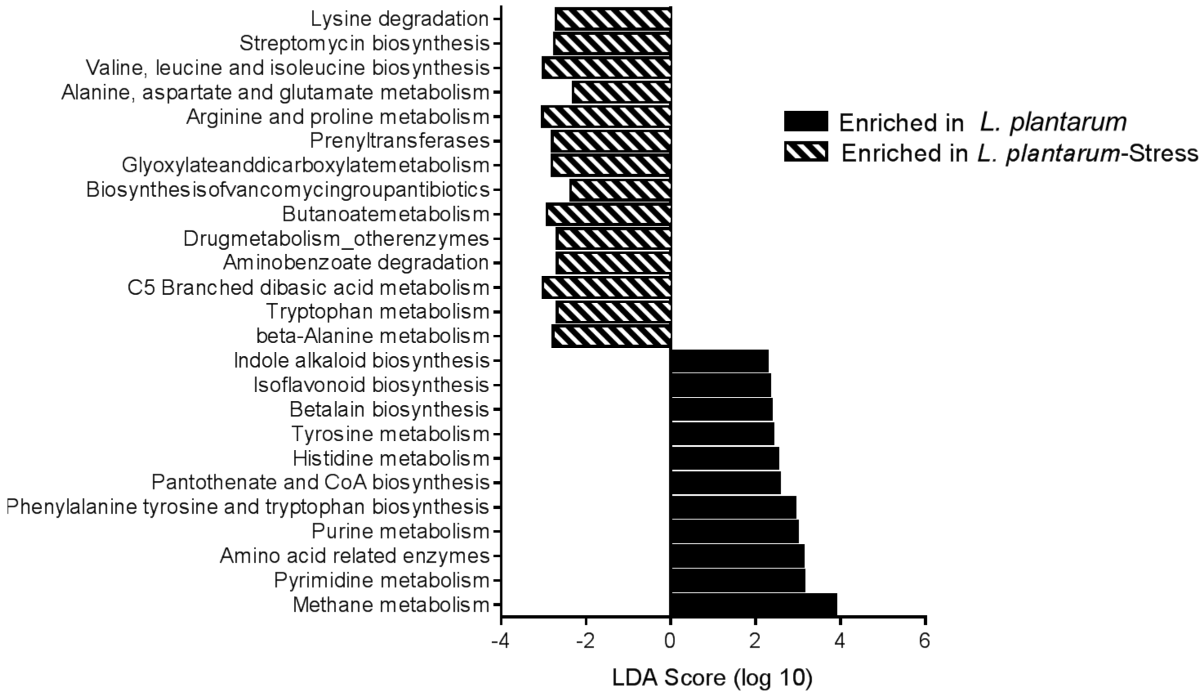

Supplementary Figure 2 **PICRUSt analysis reveals stress-induced functional alterations of the GM in *L. plantarum* treated zebrafish.** Phylogenetic Investigation of Communities by Reconstruction of Unobserved States (PICRUSt) predicted functional profile alteration of microbial communities in *L. plantarum* treated zebrafish subjected to chronic unpredictable stress (CUS). (*n* = 12 fish/group). (Linear discriminant analysis; LDA).

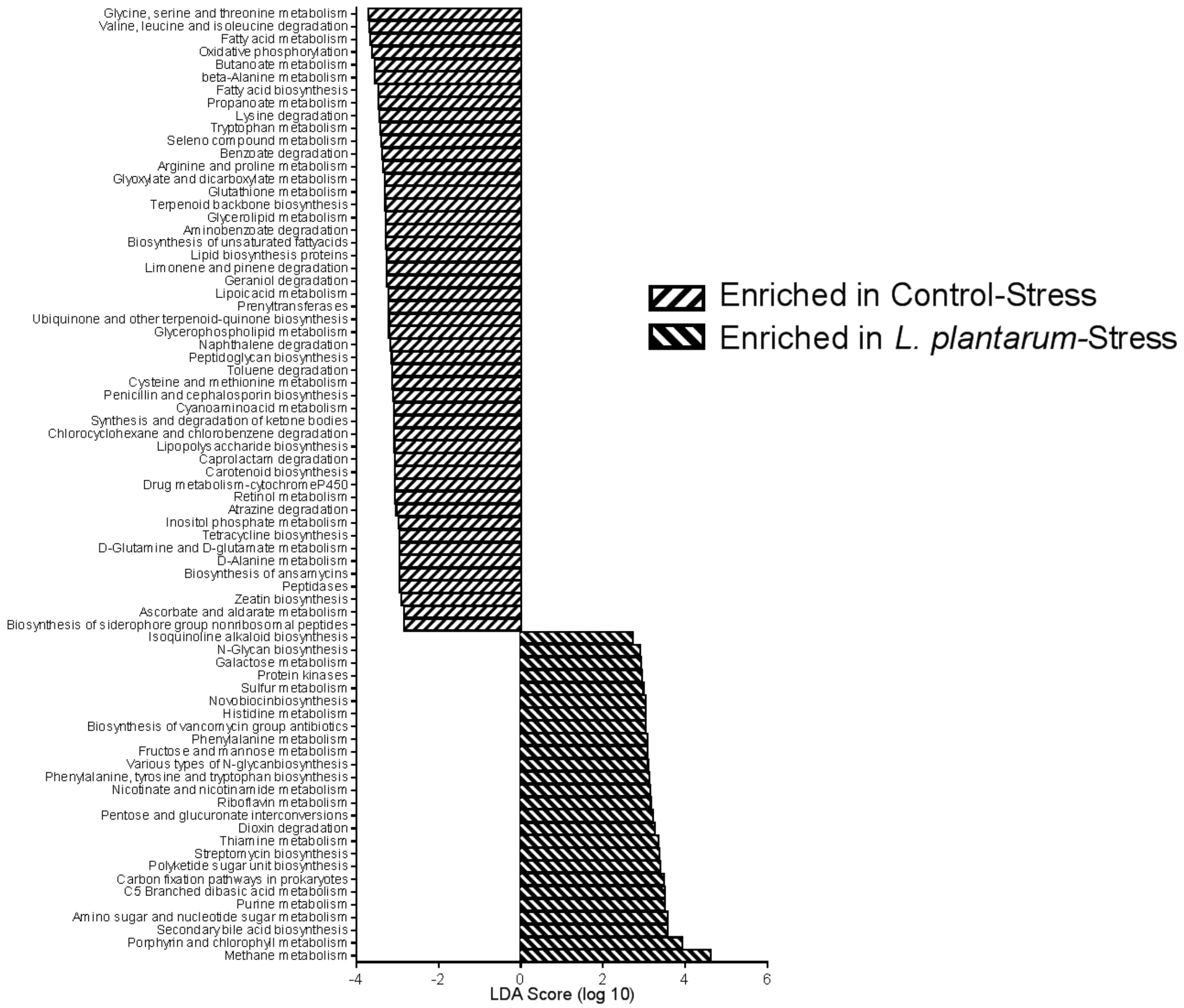

Supplementary Figure 3 **PICRUSt analysis reveals *L. plantarum* protection of up-and down- regulation of stress-induced functional alterations of the GM.** Phylogenetic Investigation of Communities by Reconstruction of Unobserved States (PICRUSt) predicted functional profile alteration of microbial communities in both control and *L. plantarum* treated zebrafish subjected to chronic unpredictable stress (CUS). (*n* = 12 fish/group). (Linear discriminant analysis; LDA).

	Morning	Evening
**Day 1**	Chasing (8 minutes)	Tank changes (x6)
**Day 2**	Over-crowding (60 minutes)	Dorsal body exposure (2 minutes)
**Day 3**	Social isolation (30 minutes)	Tank changes (x6)
**Day 4**	Dorsal body exposure (2 minutes)	Chasing (8 minutes)
**Day 5**	Tank changes (x6)	Dorsal body exposure (2 minutes)

Supplementary Table 3 **Chronic unpredictable stress (CUS) paradigm.**

Gene Symbol	Forward Primer	Reverse Primer	Reference
gad1	5'-AACTCAGGCGATTGTTGCAT-3'	5'-TGAGGACATTTCCAGCCTTC-3'	*Hortopan et al (2010)*
gabra1	5'-TCAGGCAGAGCTGGAAGGAT-3'	5'-TGCCGTTGTGGAAGAACGT-3'	*Hortopan et al (2010)*
slc6a4a	5'-TAACCACTACAGTTTGGCTTGATG-3'	5'-AACAGTTAACCGAGCTTGTGAT-3'	*Wong et al (2013)*
slc6a4b	5'-GCCGAGGAGTTTTACACGAGGA-3'	5'-ACATATGGCAGGGTGGCAGT-3'	*Wong et al (2013)*
oxtl	5'-ATTCGACAGTGTATGCCGTG-3'	5'-TCACACGGAGAAGGGAGAAA-3'	*Wong et al (2013)*
npy	5'-ACTACATCAACCTCATAACAAGGC-3'	5'-GATGAGATCACCATGCCAAATGAT-3'	*Wong et al (2013)*
ef1a	5'-CCTCTTGGTCGCTTTGC-3'	5'-GGTGTGATTGAGGGAAATTCA-3'	*Wong et al (2013)*

Supplementary Table 4 **Complete list of primer sequences used for qPCR.**
